# Neuroanatomical correlates of individual differences in the object choice task in chimpanzees (*Pan troglodytes*)

**DOI:** 10.3389/fpsyg.2022.1057722

**Published:** 2022-11-25

**Authors:** William D. Hopkins, Michele M. Mulholland, Mary Catherine Mareno, Sarah J. Neal Webb, Steven J. Schapiro

**Affiliations:** ^1^Department of Comparative Medicine, Michale E. Keeling Center for Comparative Medicine and Research, The University of Texas MD Anderson Cancer Center, Bastrop, TX, United States; ^2^Department of Experimental Medicine, University of Copenhagen, Copenhagen, Denmark

**Keywords:** brain, chimpanzee, social cognition, joint attention, object choice task

## Abstract

Declarative and imperative joint attention or joint engagement are important milestones in human infant development. These have been shown to be a significant predictor of later language development and are impaired in some individuals with, or at risk for, a diagnosis of autism spectrum disorder. Comparatively, while chimpanzees and other great apes have been reported to engage in imperative joint attention, evidence of declarative joint attention remains unclear based on existing studies. Some have suggested that differences in methods of assessing joint attention may have an influence on performance in nonhuman primates. Here, we report data on a measure of receptive joint attention (object choice task) in a sample of captive chimpanzees. Chimpanzees, as a group, performed significantly better than chance. By contrast, when considering individual performance, there was no significant difference in the number of those who passed and those who failed. Using quantitative genetic analyses, we found that performance on the object choice task was not significantly heritable nor were there any significant effects of sex, rearing history, or colony. Lastly, we found significant differences in gray matter covariation, between those who passed or failed the task. Those who passed contributed more to gray matter covariation in several brain regions within the social brain network, consistent with hypotheses regarding the importance of these regions in human and nonhuman primate social cognition.

## Introduction

An important developmental milestone in children is joint attention/engagement, a dyadic process in which preverbal individuals begin to respond to, and initiate, nonverbal bids of communication *via* the use of gaze, gesture, and vocalizations (Adamson, 1996). For example, around 4–6 months of age, typically developing children begin to respond by orienting to the communicative actions and bids by humans (usually parents and caretakers), such as following gaze cues and/or pointing responses (Tomasello, 1995; Adamson, 1996), otherwise known as receptive joint attention (RJA). Between 9 and 12 months of age, typically developing children not only respond to communicative cues, but also begin initiating them, including directing communication toward social partners, synchronizing their behavior with these partners, and responding to the communicative behaviors of others (Tomasello, 1995; Adamson, 1996; Carpenter et al., 1998). Most early communication is nonverbal (e.g., facial expressions, gaze, gestures). Typically developing children usually learn that pointing and gaze direction refer to an external referent by 12–15 months of age, and they point at and direct their attention to the referent (i.e., initiation of joint attention behavior, IJA; Carpenter et al., 1998). Many have suggested that, given the universal expression of RJA and IJA by human infants in a variety of cultures, comprehension and initiation of joint attention behaviors are the foundation for subsequent language and speech development. For example, in developmentally-typical children, the rate of language development is predicted by the age of onset of joint attention behaviors (Bates et al., 1975, 1987; Carpenter et al., 1998; Nichols et al., 2005; Whalen et al., 2006; Mundy et al., 2007). The importance of joint attention for typical socio-communicative development has been clearly established in clinical studies of children at risk for or with a diagnosis of Autism Spectrum Disorder (ASD). There is significant overlap between social and communication impairment, particularly for pre-verbal children with ASD (Landa et al., 2007). Young children at risk for ASD or diagnosed with ASD display impaired IJA and RJA abilities (Dawson et al., 2002; Presmanes et al., 2007; Ibanez et al., 2012). The neurobiological mechanisms underlying ASD are still unknown and likely involve multiple systems (Cody et al., 2002; Lotspeich et al., 2004; Herbert et al., 2005; DiCicco-Bloom et al., 2006; Hadjikhani et al., 2006; Hazlett et al., 2006; Mosconi et al., 2006; Girgis et al., 2007; Minshew and Williams, 2007; Amaral et al., 2008; Ha et al., 2015; Mundy, 2017; Pan et al., 2021); however, the potential consequences of atypical communicative and social behavior on language development and the brain are likely significant.

Some researchers have made a distinction in joint attention between imperative and declarative communication, particularly with regard to pointing (Liszkowski et al., 2004; Tomasello, 2008). Imperative pointing, or initiating behavioral requests, reflects an individual’s request for an object or action from another social agent and is therefore instrumental in function. For example, a child pointing at a cookie as a request for another person to give them the cookie. In contrast, declarative pointing is allegedly motivated by the desire to simply share information with another individual with no explicit expectation of a response. For example, a child pointing to an airplane in the sky in order to share that information with another person. Similarly, with respect to comprehension, the distinction has been made between responding to a behavioral request or to a declarative signal. Declarative communication, and particularly pointing, likely reflects the initiation of shared joint attention, whereas initiating and responding to behavioral requests reflects different underlying cognitive, neurological, and motivational processes (Baron-Cohen, 1995; Mundy et al., 2007; Cochet and Vauclair, 2010a; Cochet and Vauclair, 2010b; Leavens, 2012; Mundy, 2017). Moreover, and critically, it has been hypothesized that declarative, rather than imperative pointing, is the more important and critical cognitive and linguistic dimension of joint attention in predicting the subsequent development of language and speech (Iverson and Goldin-Meadow, 2005; Tomasello, 2008; Liszkowski et al., 2009), as well as distinguishing children at risk for ASD (Camaioni et al., 2004), though empirical support for this hypothesis is tentative at best (Ibanez et al., 2012). Indeed, direct studies that have longitudinally assessed both imperative and declarative signaling as predictors of subsequent language development in the same subjects are somewhat limited in the literature and the findings are not entirely consistent (Colonessi et al., 2010).

### Comparative studies of joint attention

RJA and IJA are not entirely unique to humans; gaze alternation and use of communicative gestures occurs in all great ape species (bonobos, chimpanzees, orangutans, and gorillas; Halina et al., 2018), monkeys of Asia and Africa (MacLean and Hare, 2013; Meunier et al., 2013; Leavens et al., 2015), and some non-primate species (Krause et al., 2018), and is identical to the early communicative acts of human children (Bates et al., 1975; Leavens et al., 2008). Great apes have been shown to communicate intentionally, point referentially, and initiate joint attention during both intra- and interspecies communication (Pika et al., 2003, 2005; Liebal et al., 2004, 2005; Cartmill and Byrne, 2007; Leavens et al., 2008, 2009; Pika, 2008; Leavens and Racine, 2009). Apes are known to request food items *via* gestures while gaze alternating between the referent and the social agent (Leavens et al., 1996; Leavens and Hopkins, 1998; Leavens and Hopkins, 1999; Tanner and Byrne, 2010; Gretscher et al., 2017) in a manner very similar to the IJA progression of developing human children (Bates et al., 1975, 1987; Lock, 2001; Abbot-Smith et al., 2004; Kirchhofer et al., 2012; Hopkins et al., 2013; Tempelmann et al., 2013). Though it is generally recognized that great apes will request foods that are otherwise unattainable or use imperative pointing, it has been hypothesized that great apes do not initiate declarative pointing, nor do they respond reliably to declarative (or imperative) cues (Tomasello, 1996; Tomasello and Camaioni, 1997; Liszkowski et al., 2004; Tomasello, 2008; Tempelmann et al., 2013). The primary data in support of this claim comes from studies on the object choice (OC) task (Mulcahy and Hedge, 2012). In the OC task, two or more opaque cups are baited with food out of view of the subject. At the start of the trial, a human experimenter uses gaze, pointing cues, or vocalizations (or some combination of these cues) to indicate to the ape subject which cup to choose in order to get the reward. Though some early studies reported that apes were incapable of correctly responding on the OC task, several recent review articles have shown that performance on the OC task by great apes and other species is influenced by a number of situational and procedural factors (Mulcahy and Hedge, 2012; Caspar et al., 2018; Clark et al., 2019). Tomasello and Carpenter (2007) and Call (2009) have suggested that one possibility for the putative lack of declarative pointing abilities in apes and other animals is that, though they may read the attention of other species, they do not read their mind or possess the notion of shared intentionality.

In the current study, one aim was to examine the influence of genetic and experiential factors on OC task performance in chimpanzees. Specifically, because we have extensive pedigree and life history information on the captive populations of chimpanzees in this study, we used quantitative genetic analyses to test for (Adamson, 1996) heritability in performance on the OC task and (Tomasello, 1995) the influence of sex and rearing history on performance. It has recently been reported that performance on a version of the OC task is significantly heritable in retriever dogs (Bray et al., 2021). Furthermore, previous studies in chimpanzees have reported significant heritability in two other tasks that purportedly assess RJA (Hopkins et al., 2014; Hopkins and Latzman, 2021). Thus, we hypothesized that if OC task performance has a genetic component, then significant heritability would be found. Regarding rearing history, previous studies have shown that both canines and apes with extensive human socialization or contact perform better on the OC task than individuals with less of these experiences. Because humans are the individuals performing the OC testing, the hypothesis is that the increased socialization or rearing experiences with humans biases performance toward these individual apes. A subset of the chimpanzees in this study were raised in a human nursery setting (see below); thus, comparing their performance to individuals raised by conspecifics allowed for comparison of these rearing experiences on performance.

In the second aim, we tested for associations between OC task performance and gray matter covariation in a subset of the chimpanzees using sourced-based morphometry. Source-based morphometry (SBM) is a relatively new approach for studying structural magnetic resonance images incorporating an independent component analysis to create spatial components that covary in gray or white matter across the entire brain. Within the SBM analysis, weighted scores are produced for each subject that reflect their contribution to the creation of each spatial component. In humans, SBM has been used to examine structural variation across the lifespan, and between clinical and non-clinical populations (e.g., Xu et al., 2009; Hafkemeijer et al., 2014; Rektorova et al., 2014; Grecucci et al., 2016; Bergsland et al., 2018). SBM has also been used to investigate structural covariation in gray matter among nonhuman species. For example, SBM has been used to examine the differences in gray matter covariation between chimpanzees with different rearing histories (Bard and Hopkins, 2018) or with different genotypes (Mulholland et al., 2020), and to investigate the relationship between structural variation and the behavioral specialization of 33 different dog breeds (Hecht et al., 2019). In this study, we used SBM to test for associations between OC task performance and gray matter covariation. Specifically, Mundy and Newell (2007), and others (Benga, 2005), have offered a proposed neurological system that underlies both receptive and productive imperative and declarative signaling in developing children. With respect to the initiation of joint attention, they define this as the anterior attention system that includes the frontal eye fields and dorsal/medial prefrontal cortex (Brodmann areas 8 and 9), orbital and ventrolateral gyri (Brodmann areas 11, 47) and anterior cingulate cortex (Brodmann area 24). In contrast, for receptive joint attention, Mundy and Newell have proposed the posterior attention system and this includes portions of the parietal lobe, such as the supramarginal and angular gyri (Brodmann areas 7, 39, 40) and the posterior superior temporal gyrus (Brodmann areas 22, 41, and 42). We hypothesized that if OC task performance in chimpanzees is associated with gray matter in brain regions comprising the posterior attention system, then chimpanzees that performed better would show higher gray matter covariation in SBM components that included the posterior temporal gyrus and parietal areas compared to apes that performed more poorly.

## Materials and methods

### Subjects

We behaviorally tested 138 chimpanzees from two samples of captive chimpanzees. One cohort was housed at the Emory National Primate Research Center (ENPRC, formally Yerkes; *n* = 65) and the other at the National Center for Chimpanzee Care (NCCC), which is part of the MD Anderson Cancer Center (*n* = 73). Within the total sample, there were 47 males and 91 females ranging in age was 9 to 51 years and, by way of rearing history, there were 68 mother-reared, 46 nursery-reared and 24 wild-born individuals. We defined a nursery-reared (NR) chimpanzee as an individual that was separated from his or her mother within the first 30 days of life due to unresponsive care, injury, or illness (see 82, 83 for details). These chimpanzees were placed in incubators, fed standard human infant formula [not supplemented with docosahexaenoic acid (DHA, an omega-3 fatty acid) as far as we know] and cared for by humans until they could sufficiently care for themselves. They were then placed with other infants of the same age until they were 3 years old (Bard et al., 1992; Bard, 1994). At 3 years of age, the nursery-reared chimpanzees were integrated into larger social groups of adult and sub-adult chimpanzees. Mother-reared (MR) chimpanzees were not separated from their mother during at least the first 2.5 years of life and were raised in ‘nuclear’ family groups of conspecifics, ranging in size from 4 to 20 individuals. Wild-born (WB) apes were individuals brought to the United States prior to the 1974 CITES ban on importation of chimpanzees from the wild. Within the sample of 138 chimpanzees that were tested on the OC task, archival structural magnetic resonance images (sMRI) were available for 118 individuals. The average time between behavioral testing and scanning was 5.37 years (SD = 4.16). All aspects of this research conformed to existing US and NIH federal policies on the ethical use of chimpanzees in research and was approved by the Institutional Animal Care and Use Committees at both NCCC and YNPRC.

### Object choice task procedure

All subjects were tested in the indoor or outdoor portions of their home enclosures at each facility between 9:00 AM and 6:00 PM. The individuals testing the chimpanzees on the OC task were very familiar with each chimpanzee and had interacted or worked with them for a minimum of 5 years prior to data collection. The OC task was administered following the methods described in Lyn et al. (2010). Briefly, we created paper tubes (with one end closed) that we could bait with a grape. When presented, two tubes were placed on the right and left sides of a testing table (equidistant from the center), with only one of the tubes baited. The baited side was randomly determined prior to data collection. Before testing, the chimpanzees were trained on the task so that they would learn to select the tube with the food reward [see Lyn et al., 2010 for training procedures]. Each chimpanzee received one session of 24 test trials across three declarative conditions (point, vocalize, and point/vocalize combined) presented in random order. In the point condition, the experimenter pointed (full arm and index finger extended) at the baited tube and stated “[Subject name], the food is in here” then alternated their gaze between the subject and the baited tube. In the vocalize condition, the experimenter leaned toward the baited tube while simulating chimpanzee food grunts and alternated their gaze between the subject and the baited tube. In the combined condition, the experimenter simulated food grunts and pointed at the baited tube, alternated their gaze between the subject and the baited tube, and stated “[Subject name], the food is in here.” In all trials, the baiting of the tube was done out of the subject’s view, and a correct response was recorded if the subject chose the baited tube.

### Behavioral data and heritability analyses

Performance on the OC task was quantified two ways. First, the primary dependent measure was the percentage of responses across the 24 test trials for each subject. In addition, we computed binomial *z*-scores for each subject to evaluate whether individual performance was significantly better than chance (50% correct). Subjects with a binomial *z*-score ≥ 1.96 were classified as passing the test while all others were classified as failing. Both inferential (analysis of variance, *t*-tests) and non-parametric statistics (e.g., Chi-square) were used to test for the effects of sex, rearing history, and colony on performance. For all tests, alpha was set to *p* < 0.05, and post-hoc tests were performed using Tukey’s HSD, when needed.

We used the SOLAR (Sequential Oligogenic Linkage Analysis Routines) software package to estimate the heritability of object choice performance considering the entire chimpanzee pedigree. Specifically, the identification codes of offspring, dam and sire (when known) dating as far back as possible into the inception of each chimpanzee colony were entered into a file as well as their sex and rearing history. These data were then imported into SOLAR to create the pedigree structure. The pedigree structure of the entire chimpanzee sample has been published elsewhere (Hopkins et al., 2015) with some animals being fourth generation related to each other. We used a polygenic model that estimated the influence of additive genetic variation (based on the pedigree) and the covariates, calculated heritability and its associated value of *p*, as well as the proportion of variance accounted for by the final covariates included in the model. The significance level for heritability was set at *p* ≤ 0.05.

### Neuroimaging data and SBM analysis

The method of magnetic resonance image collection and post-image processing steps have been described in previous studies (see (Hopkins et al., 2020; Mulholland et al., 2020) for details). Briefly, sMRI scans were collected on either a 1.5 T (*n* = 57) or 3 T (*n* = 47) scanner from chimpanzees during their annual physical examinations. The sMRI scans were subsequently resampled at 0.625 mm isotropic resolution, aligned in the AC-PC axis, skull stripped using the BET function in FSL (Smith, 2002; Jenkinson et al., 2005), N4 bias corrected in 3DSlicer^1^ (Boyes et al., 2008; Tustison and Gee, 2010; Tustison et al., 2010; Tustison and Avants, 2010; Tustison et al., 2014) and denoised using the MRI Denoising Package for MATLAB (R2015b; Mathworks, Natick, Massachusetts, United States; Coupé et al., 2008). The sMRI preprocessed scans were then processed in the VBM pipeline within FSL,^2^ which included segmentation, creation of a study-specific template and subsequent linear registration, followed by non-linear registration of segmented gray matter volume to the study-specific gray matter template. The modulated gray matter volumes were then smoothed with an isotropic Gaussian kernel with a sigma of 2 mm. The smoothed, modulated gray matter volumes were then subjected to a source-based morphometry (SBM) analysis using the Group ICA of fMRI Toolbox (GIFT)^3^ in MATLAB R2015b using the default parameters for all variables. For any SBM components found to be associated with OC task performance, we thresholded the volume + 3.0 and identified those brain areas as the primary regions comprising it.

## Results

### Behavioral data and heritability

Though our main interest was in the overall performance of the chimpanzees on the OC task, recall that subjects received eight trials in each of three different cuing conditions: 1. vocal and point, 2. point alone, and 3. vocal alone. Thus, in the initial analysis, we compared performance between three conditions using a mixed-model analysis of variance (ANOVA). Cue type was the repeated measure, while sex, colony, and rearing history were between-group factors. We found a significant main effect for cuing condition *F*(2, 252) = 11.177, *p* < 0.001. *Post-hoc* analysis indicated that performance in the vocal + point (*Mean Correct Trials* = 5.649, *se* = 0.166) and point alone (*Mean Correct Trials* = 5.701, *se* = 0.175) conditions were significantly better than in the vocal alone condition (*Mean Correct Trials* = 4.740, *se* = 0.185). No other main effects or interactions were significant [sex *F*(1,126) = 0.047 *p* = 0.828; colony *F*(1,126) = 1.153 *p* = 0.285; rearing *F*(2,126) = 0.135 *p* = 0.874; all interactions *F*(2,126) = 0.003–2.131, *p* > 0.05].

When considering overall performance, there was no significant relationship between performance and age, *r*(116) = −0.054, *p* = 0.564. Using 50% correct as an estimated population mean, one-sample *t*-tests revealed that the chimpanzees performed significantly better than chance on the OC task (*Mean Percent Correct* = 66.30, se = 0.97) *t*(137) = 16.63, *p* < 0.001; however, based on their binomial *z*-score classification, a chi-square goodness-of-fit test was not significant *X*^2^(1, *N* = 138) = 2.899, *p* = 0.089 with 57% of the subjects classified as failing (OC−; *n* = 79) and 43% as passing (OC+; *n* = 59) this task. Chi-square tests of independence failed to show any significant association between OC performance classification and either sex [*Χ^2^*(1) = 0.223, *p* = 0.637], rearing history [*Χ^2^*(1) = 1.731, *p* = 0.421], or colony [*Χ^2^* = 0.847, *p* = 0.357]. Quantitative genetic analysis using SOLAR revealed that OC task performance was not significantly heritable (*H^2^* r = 0.00, *p* = 0.50) and neither sex (*p* = 0.745), rearing history (*p* = 0.109), nor colony (*p* = 0.139) were significant covariates.

### SBM results

**Figure 1:**
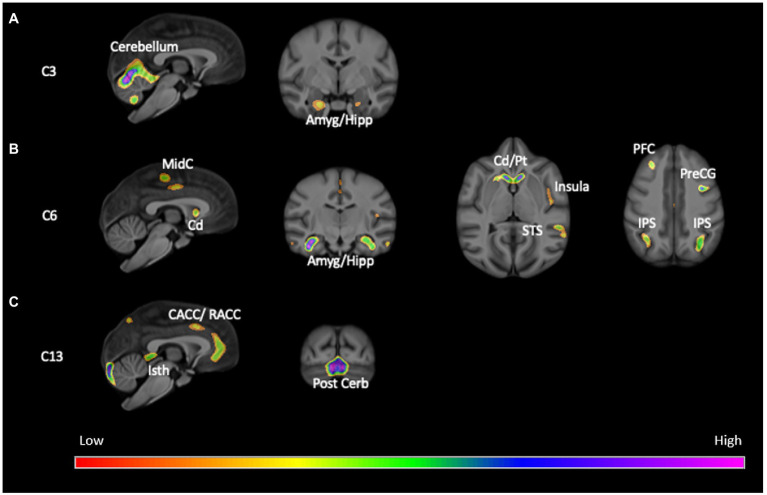
Screen shots of brains regions within **(A)** Component 3, **(B)** Component 6, and **(C)** Component 13. Cd, caudate; Pt, putamen; Amyg/Hipp, amygdala and hippocampus; insula, insula; STS, superior temporal sulcus; IPS, intraparietal sulcus; PFC, prefrontal cortex; PreCG, precentral gyrus; CACC/RACC, caudal and rostral anterior cingulate cortex; Isth, isthmus of the cingulate; MidC, mid-cingulate; Cerb, cerebellum; Post Cerb, posterior cerebellum. The colors indicate standardized z-values from the SBM component scores.

**Figure 2:**
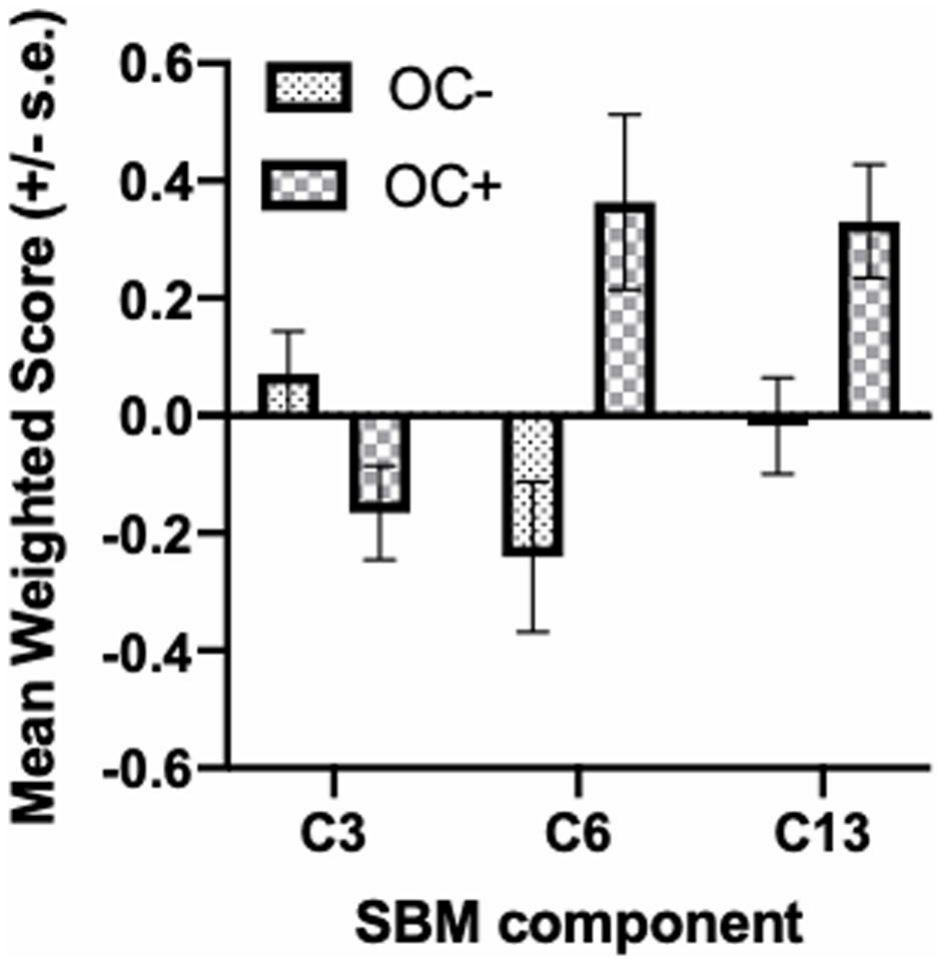
Mean SBM weighted score (+/− s.e.) in OC− and OC+ apes for component 3, 6, and 13.

The SBM analysis on the 118 chimpanzees on which OC task performance data was available revealed 13 components for this cohort of chimpanzees. We compared the weighted scores for these 13 components between chimpanzees classified as passing (OC+) or failing (OC−) the object choice task using a repeated measures analysis of covariance. The 13 weighted component scores served as the repeated measures while sex (Male, Female) and OC task performance classification (OC+, OC−) were the between group factors. The difference in age of the chimpanzees between the collection of the sMRI scans and OC testing, and scanner magnet were covariates. A significant interaction between OC group and component was found *F*(12, 1,344) = 2.110, *p* = 0.014. Subsequent post-hoc analysis revealed that OC+ had significantly higher weighted scores than OC− apes for components 6 and 13 (see Figure 1). Component 6 was comprised of the dorsal prefrontal cortex (right hemisphere), precentral gyrus (left), amygdala/hippocampus (bilateral), caudate (bilateral), putamen (right hemisphere), insula (left hemisphere), mid-cingulate (bilateral), middle temporal sulci (bilateral), intraparietal sulcus (bilateral) and superior temporal sulci (left hemisphere). Component 13 included the cerebellum (bilateral) and amygdala/hippocampus (bilateral). By contrast, for component 3, OC+ apes had lower weighted scores compared to the OC− apes (Figure 2). Volumes for each brain region within components 3, 6, and 13 are shown in Table 1. Partial correlations between the component weighted scores and age at MRI acquisition while controlling for relatedness, sex, rearing, and scanner, revealed no significant relationship between age and components 3 [*r*(112) = 0.050 *p* = 0.597], 6 [*r*(112) = −0.002, *p* = 0.981], and 13 [*r*(112) = −0.078, *p* = 0.408].

**Table 1:** Brain region gray matter volumes for SBM components 3, 6, and 13.

Brain region	Volume (L/R)
**Component 3**	
Cerebellum	11835.21
Amygdala/hippocampus	253.42/711.43
**Component 6**	
Caudate	518.31/607.67
Putamen	0.00/140.14
Insula	539.06/0.00
Superior temporal sulcus	469.73/0.00
Middle temporal sulcus	410.40/409.18
Amygdala/hippocampus	870.61/992.68
Dorsal prefrontal cortex	0.00/277.10
Precentral gyrus	312.26/0.00
Middle cingulate sulcus/gyrus	538.23
Intraparietal sulcus	479.98/325.44
Lateral cerebellum	0.00/323.49
**Component 13**	
Caudal/rostral cingulate	1691.16
Posterior cerebellum	1806.88
Medial temporal pole	533.45/759.52
Isthmus of the cingulate	292.97

Values are in mm^3^.

## Discussion

The results of this study can be summarized as follows: First, when considered as a collective group, chimpanzees scored significantly higher than chance on the OC task; however, when evaluating individual performance based on binomial *z*-scores, there was no significant difference in the number of OC+ (passing) and OC− (failing). Second, OC task performance was not significantly heritable nor were there any significant effects of sex, rearing history, or colony. Lastly, significant differences in gray matter covariation were found between chimpanzees that passed or failed the OC task.

Many have reported that chimpanzees fail tasks that require responding to, or the production of, declarative signals, particularly for the OC task (Mulcahy and Hedge, 2012; Clark et al., 2019). That is to say, chimpanzees, as a species, are allegedly poor at or incapable of comprehending or producing declarative communicative signals. Consistent with the meta-analysis reported by Clark et al. (2019), the results reported here challenge this claim; however, this inference depends, in part, on the interpretation of the group compared to individual results. Notably, consistent with nearly every other report in the literature on OC task performance (Clark et al., 2019), though the average performance of the chimpanzees was significantly better than chance based on the one sample *t*-test, there was no statistical difference between the number of OC+ and OC− chimpanzees. In our view, the dissociation in interpretation of performance based on the group and individual levels of analysis is not a trivial issue.

There are numerous studies in the literature on animal social cognition that utilize group averages as a basis for inferring either (Adamson, 1996) species differences in performance or (Tomasello, 1995) group or species level capacities for a given psychological trait (Karg et al., 2015; Joly et al., 2017). As argued elsewhere (Hopkins and Sherwood, 2022), one problem in using group average data to evaluate species-specific capacities or species differences in performance is that, within a given sample of subjects, it is feasible that the group as a whole can perform significantly better than chance, while no individual may perform the task significantly better than chance. As an example, 8 chimpanzees could be administered 24 trials on the OC task and perform at 50%, 52%, 53%, 55%, 55%, 58%, 62%, and 67% correct. A one-sample *t*-test on these data reveals that the chimpanzees, as a group, perform statistically better than chance *t*(7) = 3.265, *p* = 0.014 while not a single individual ape performed better than chance based on their binomial *z*-scores. This is particularly true in studies where the subjects are administered very few trials, which can often be the case in experiments of cognition in human and nonhuman primates (Buttelmann et al., 2007; Herrmann et al., 2007, 2010; Russell et al., 2011; Schmitt et al., 2011; Karg et al., 2015; Rosati et al., 2016; Krupenye and Hare, 2018; Bettle and Rosati, 2019). In short, we believe that, in addition to group averages, there is also a need to report individual performance data in studies of animal cognition (including humans; Herrmann et al., 2006). This would increase the rigor of these studies and likely improve their repeatability and replication across laboratories and settings.

The findings reported here also failed to find any significant sex or rearing history effects on OC task performance. Further, OC task performance was not significantly heritable. These findings differ from previous reports on OC task performance among apes in two ways. First, regarding rearing history, Lyn et al. (2010) and Call and Tomasello (1994) reported that human enculturated apes perform significantly better than age-sex matched controls who were not human enculturated. Recall that a portion of our chimpanzees were raised by humans in a nursery setting for the first 3 years of life which some might suggests would constitute human enculturation. However, the human enculturated apes tested by Benga (2005)) and Call and Tomasello (1994) were individuals with extensive, life-long rearing histories, including training on alternative communication systems with humans which differs substantially from the NR chimpanzees in this report. Therefore, it appears that rearing of chimpanzees by humans in a nursery setting does not provide the level of experience or stimulation to enhance their OC task performance over apes raised by conspecifics. Second, performance on the OC task has been reported to be significantly heritable in canines (largely retriever breeds; Bray et al., 2021), a finding that differs from our results in chimpanzees. Because canines have been domesticated and the subjects were purebred animals, one interpretation of this difference may be that the selective breeding for traits strongly expressed in retriever canines facilitated the processing of human communicative cues in some canine species. This explanation is certainly possible but performance on the OC task varies substantially among different breeds of canines, as well as between pet dogs compared to individuals living in shelters or other environments (Udell et al., 2010; Clark et al., 2019). Thus, it is possible that selective breeding for certain traits manifest in retriever canine breeds may account for the reported heritability but, in the absence of data from non-retriever breeds, it is not possible to attribute these results to a broader genetic effect attributable to domestication.

Regarding the SBM results, OC+ chimpanzees contributed more to the creation of two gray matter components that included a number of brain regions within the social brain network including the amygdala, hippocampus, insula and middle and posterior superior temporal gyrus (Adolphs, 2009; Kennedy and Adolphs, 2013; see Figure 1). These findings are consistent with the Mundy and Newell hypothesis regarding the brain regions implicated in the posterior attentional network and corroborate previous findings in chimpanzees of an association between gray matter volume in the superior temporal gyrus and a different measure of RJA. The findings are also consistent with hypotheses regarding the role of the middle and superior temporal gyrus and sulcus in the processing and interpretation of social stimuli (Redcay, 2008; Redcay et al., 2012). OC+ chimpanzees contributed less to component 3 compared to the OC− apes. Interestingly, the largest brain region within component 3 was the rostral and caudal anterior cingulate, a brain region hypothesized to be part of the anterior attention network and hypothesized to be linked to the initiation of imperative and declarative signals (Benga, 2005; Mundy and Newell, 2007). Previous studies have reported significant associations between gray matter volume within the anterior cingulate in chimpanzees(Hopkins and Taglialatela, 2012), suggesting that there is some dissociation in the brain regions associated with initiation of and responding to RJA cues.

The shared intentionality hypothesis proposes that increased selection for cooperative and prosocial behavior led to the emergence of the ability for shared intentions between interlocutors, and manifests in a variety of advanced, human-specific cognitive abilities, such as triadic joint attention, declarative communication, imitative learning and teaching (Tomasello, 1999). It has been correctly recognized that there is some evidence that apes raised by humans in complex socio-linguistic environments do engage in triadic joint attention and declarative pointing (Leavens and Bard, 2011). Indeed, previous research has shown that bonobos and chimpanzees that have been raised by humans in a socio-communicative linguistic environment, much like human infants, perform significantly better on the OC task than apes raised in standard captive settings by humans or conspecifics (Lyn et al., 2010). Similarly, among language-trained apes that communicate with humans *via* visual-graphic symbols, between 10% and 15% of their keyboard utterances have been characterized as declarative in function (Lyn et al., 2011). Though this percentage was much lower than data from 15-month-old human children, it does suggest that the differences in declarative pointing ability between humans and apes are not absolute, but rather quantitative and potentially malleable, as a function of the early social rearing experience of the subjects.

Importantly, as pointed out by Call (2009), evidence of declarative pointing in language-trained apes begs the question of why non-language-trained apes do not use these presumed innate abilities in the wild or captivity? We would offer two possible explanations. First, evidence from this study suggests that performance on the OC task is not heritable; thus, the assumption that this is an inherent, innate ability that is presumably under some genetic control is not supported in this study, and, indeed, we know of no studies that have explicitly demonstrated a genetic basis for joint attention in humans. It may be the case that social learning or other experiential factors explain the development of RJA in humans and that a similar explanation might elucidate the performance of the enculturated apes compared to standard mother- or nursery-reared individuals; i.e., non-language-trained apes likely experience less responsive caregiving environments than language-trained and other enculturated apes(Bard et al., 2014). That is to say, attempts to compare declarative communication abilities between human children and nonhuman primates is exceedingly difficult without controlling for the extent of reinforcement history and learning experiences that apply to each species’ experiences prior to testing.

Second, the capacity for declarative communication may be present in chimpanzees and perhaps, many other primate species, but it manifests itself differently than the communicative behaviors of typically developing preverbal children. For instance, recent studies in wild chimpanzees have shown that they will produce alarm calls in the presence of a predator (harmful snake) more frequently when the chimpanzees accompanying the focal subjects are unaware or ignorant of the presence of the snake (Crockford et al., 2012; Schel et al., 2013). Chimpanzees are less likely to produce the alarm calls when the accompanying chimpanzees are aware of the presence of the snake. Unlike the production of alarm calls in response to predators in other nonhuman primates, such as the well-known case of vervet monkeys (Seyfarth et al., 1980; Seyfarth and Cheney, 1993; Seyfarth and Cheney, 2010), the chimpanzees appear to be “informing” other group members of the presence of the snake, rather than simply responding fearfully, to which other group members respond (Seyfarth and Cheney, 2010). By definition, this constitutes a form of declarative communication; however, this is a vocal signal, not a manual pointing response, which is the primary behavioral measure investigators have used to characterize declarative communication in other studies of apes and children. Similarly, in some studies with children, the subjects are presented with novel objects in the presence of a caretaker and the researchers record the amount of time the child spends showing the object or the number of alternations in gaze the child makes between the object and the caregiver. This type of communicative behavior, which primarily manifests in gaze behavior, is considered declarative in function by some (Mundy et al., 2007) and defined as triadic joint attention by others (Carpenter et al., 1995). In either case, the overt behaviors measured are not a manual pointing response, which is again, the primary basis by which declarative communication has been measured in nonhuman apes. As Bates and her colleagues observed (Bates et al., 1975) in their paper introducing what they called the protodeclarative aspects of infant preverbal communication, pointing is simply one of a number of such communicative acts, including exhibition of self, showing of objects to others, and giving objects to others. All of these declarative behaviors are well-described in the communicative interactions of chimpanzees (Plooij, 1978; Zamma, 2002; Silk et al., 2013). In our view, an arguably less narrow, species-centric approach to defining declarative communication may provide a more valuable conceptual and methodological framework for comparative tests of the shared intentionality hypothesis.

This study is not without limitations. First, though we statistically controlled for the age of the chimpanzees when they were tested on the OC task and when they were scanned, the collection of these two data sets was spread over multiple years and therefore were not necessarily obtained in close temporal proximity to one another. Indeed, though all but one chimpanzee was tested on the OC task after the collection of the sMRI scan, the average time between behavioral testing and scanning was 5.37 years with no significant difference between OC+ *M* = 5.27 *SD* = 4.00 and OC− *M* = 5.45 *SD* = 4.29, *t*(116) = −0.235 *p* = 0.814. In light of the fact that there are age-related changes in gray matter covariation among chimpanzees (Hopkins et al., 2019; Mulholland et al., 2021), ideally the behavioral and sMRI data would have been collected at approximately the same time. Unfortunately, this is not possible, as MRI scans can no longer be acquired from chimpanzees for research purposes. Second, most if not all of the chimpanzees in this study have been subjects in previous studies on joint attention and related tasks assessing social cognition (Whiten et al., 2007; Hopper et al., 2008; Taglialatela et al., 2012; Hopkins et al., 2014; Brosnan et al., 2015; Leavens et al., 2015; Hopkins et al., 2020). It is possible that testing experiences on these previous tasks may have benefitted or hindered performance on the OC task and we cannot rule this out as a source of some of the individual variation in performance.

In summary, consistent with previous reports, the overall results demonstrate that chimpanzees comprehend declarative communication signals as measured by the object choice task, though there is considerable individual variability in performance with a significant number of apes performing below chance. OC task performance was not significantly heritable nor were there any significant effects of sex, colony, or rearing history. OC task performance was associated with gray matter covariation in the middle and superior temporal cortex, and a number of other regions, a finding consistent with the hypothesized role of these specific brain areas in RJA, and more broadly, social cognition.

## Data availability statement

The raw data supporting the conclusions of this article will be made available by the authors, without undue reservation.

## Ethics statement

The animal study was reviewed and approved by the Animal Care and Use Committee of Emory University and MD Anderson Cancer Center.

## Author contributions

WH designed the work, analyzed and interpreted the data, and wrote the initial drafts. MMu analyzed the data and wrote the initial drafts. SS, MMa, and SW acquired data. All authors contributed to the article and approved the submitted version.

## Funding

This work was supported, in part, by NIH grants AG-067419, NS-073134, NS-042867, and NS-092988. Chimpanzee maintenance at the National Center for Chimpanzee Care was previously funded by NIH/NCRR U42-OD-011197.

## Conflict of interest

The authors declare that the research was conducted in the absence of any commercial or financial relationships that could be construed as a potential conflict of interest.

## Publisher’s note

All claims expressed in this article are solely those of the authors and do not necessarily represent those of their affiliated organizations, or those of the publisher, the editors and the reviewers. Any product that may be evaluated in this article, or claim that may be made by its manufacturer, is not guaranteed or endorsed by the publisher.

## Footnotes

^1^ www.3Dslicer.org

^2^ http://fsl.fmrib.ox.ac.uk/fsl/fslwiki/FSLVBM

^3^ http://icatb.sourceforge.net
